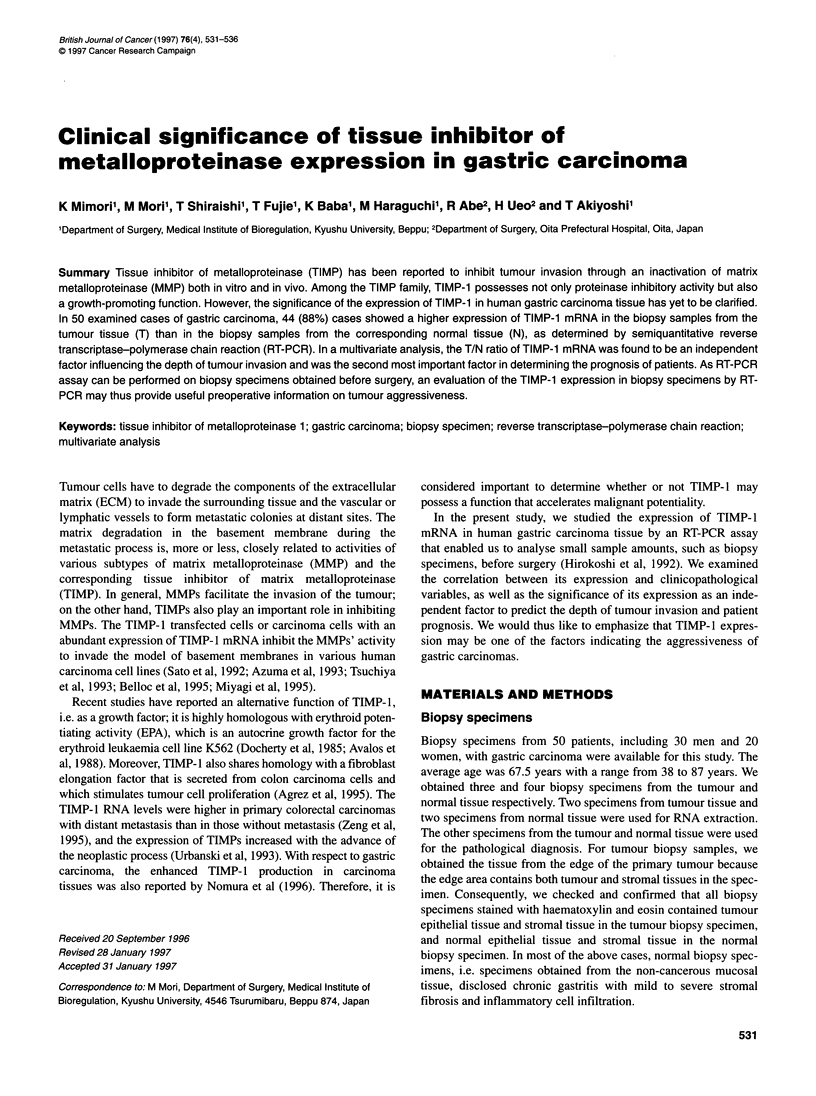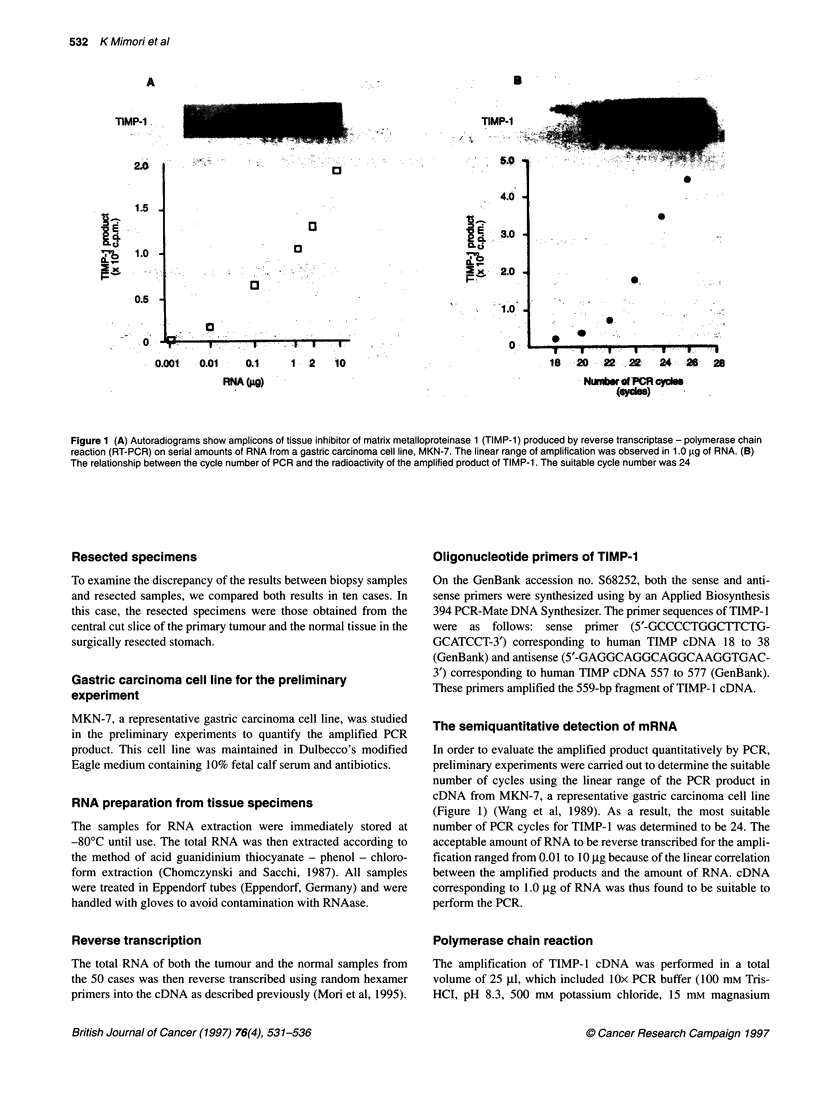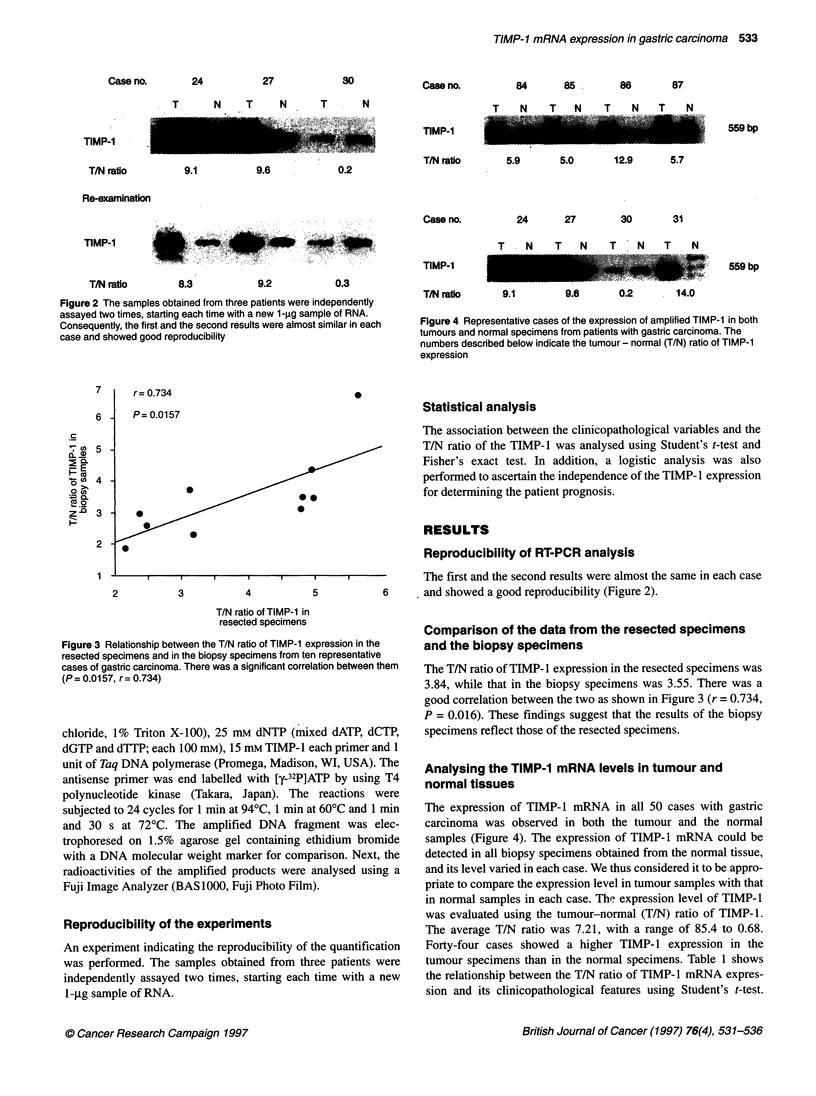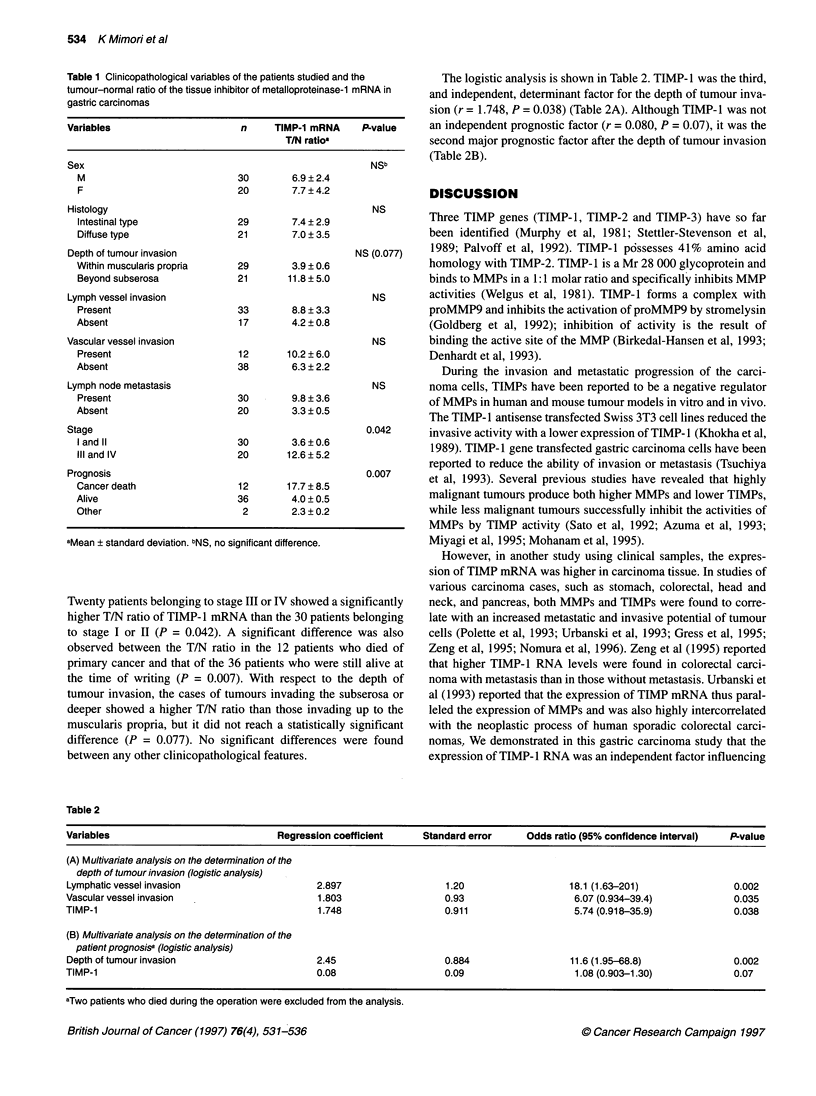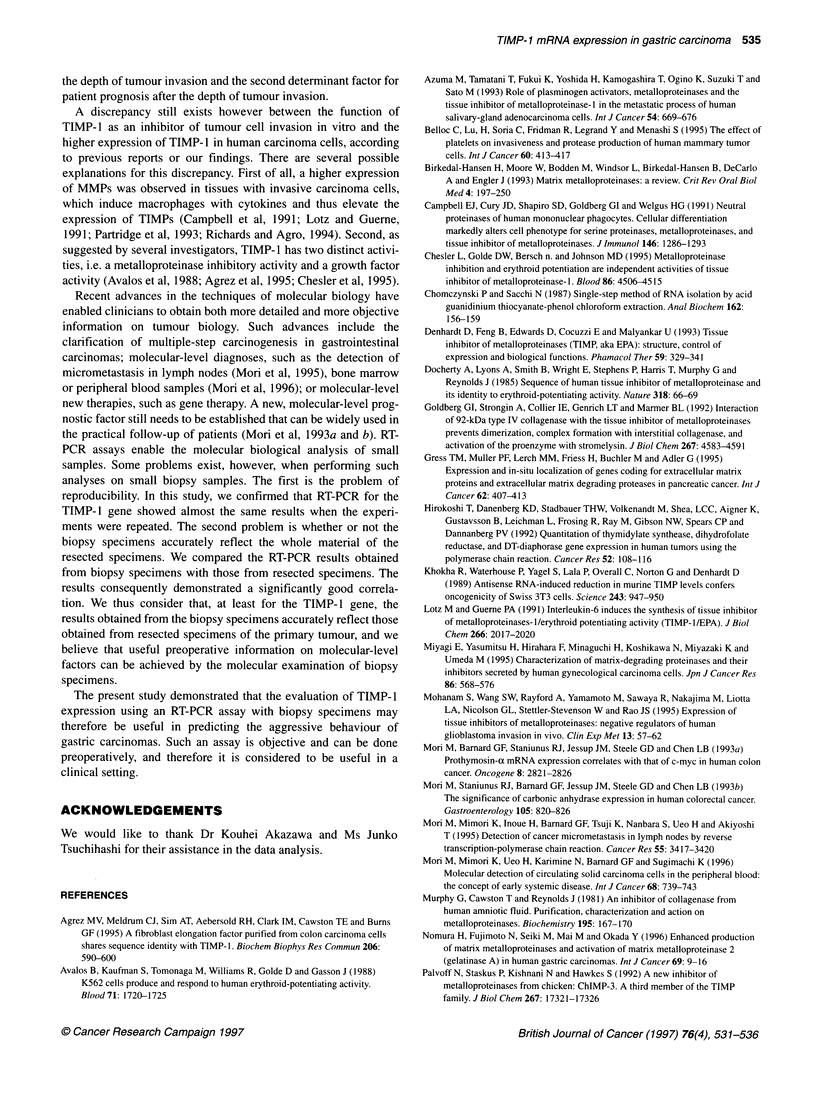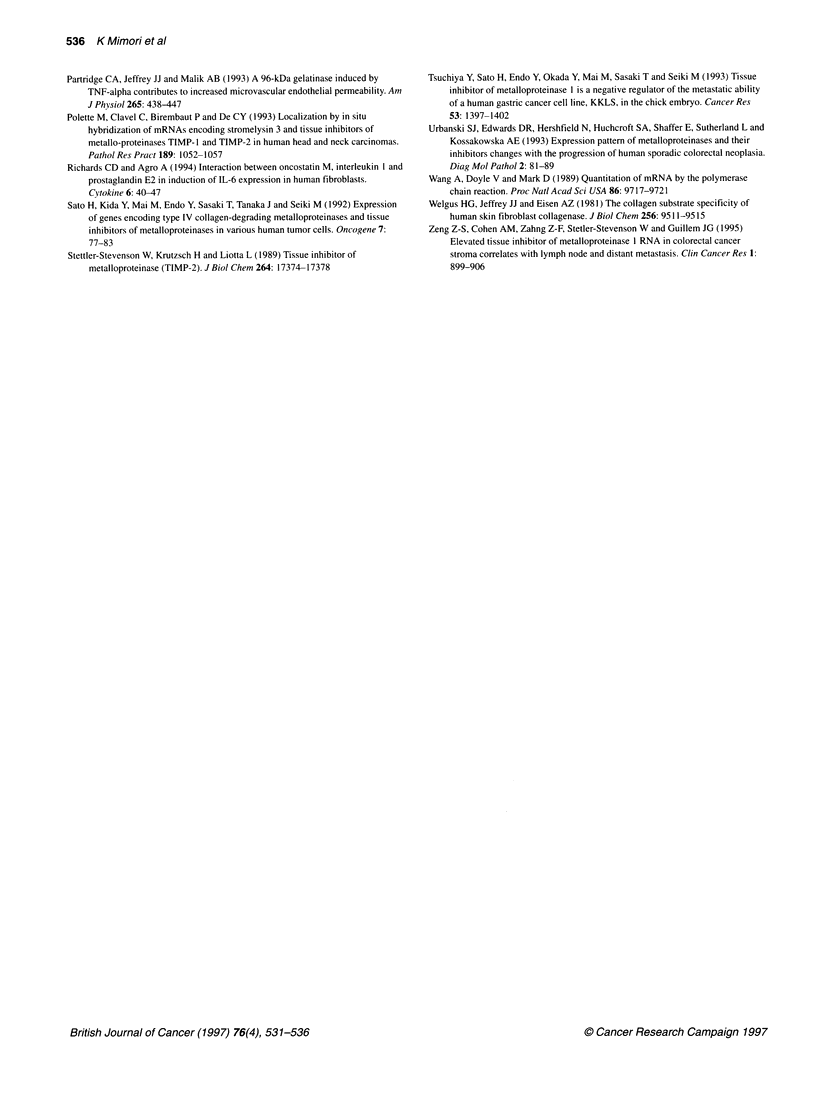# Clinical significance of tissue inhibitor of metalloproteinase expression in gastric carcinoma.

**DOI:** 10.1038/bjc.1997.420

**Published:** 1997

**Authors:** K. Mimori, M. Mori, T. Shiraishi, T. Fujie, K. Baba, M. Haraguchi, R. Abe, H. Ueo, T. Akiyoshi

**Affiliations:** Department of Surgery, Medical Institute of Bioregulation, Kyushu University, Beppu, Japan.

## Abstract

**Images:**


British Journal of Cancer (1997) 76(4), 531–536
© 1997 Cancer Research Campaign

### Clinical significance of tissue inhibitor of metalloproteinase expression in gastric carcinoma

**K Mimori[1], M Mori[1], T Shiraishi[1], T Fujie[1], K Baba[1], M Haraguchi[1], R Abe[2], H Ueo[2] and T Akiyoshi[1]**

[1]Department of Surgery, Medical Institute of Bioregulation, Kyushu University, Beppu; [2]Department of Surgery, Oita Prefectural Hospital, Oita, Japan

**Summary** Tissue inhibitor of metalloproteinase (TIMP) has been reported to inhibit tumour invasion through an inactivation of matrix metalloproteinase (MMP) both in vitro and in vivo. Among the TIMP family, TIMP-1 possesses not only proteinase inhibitory activity but also a growth-promoting function. However, the significance of the expression of TIMP-1 in human gastric carcinoma tissue has yet to be clarified. In 50 examined cases of gastric carcinoma, 44 (88%) cases showed a higher expression of TIMP-1 mRNA in the biopsy samples from the tumour tissue (T) than in the biopsy samples from the corresponding normal tissue (N), as determined by semiquantitative reverse transcriptase–polymerase chain reaction (RT-PCR). In a multivariate analysis, the T/N ratio of TIMP-1 mRNA was found to be an independent factor influencing the depth of tumour invasion and was the second most important factor in determining the prognosis of patients. As RT-PCR assay can be performed on biopsy specimens obtained before surgery, an evaluation of the TIMP-1 expression in biopsy specimens by RT-PCR may thus provide useful preoperative information on tumour aggressiveness.

**Keywords:** tissue inhibitor of metalloproteinase 1; gastric carcinoma; biopsy specimen; reverse transcriptase–polymerase chain reaction; multivariate analysis

Tumour cells have to degrade the components of the extracellular matrix (ECM) to invade the surrounding tissue and the vascular or lymphatic vessels to form metastatic colonies at distant sites. The matrix degradation in the basement membrane during the metastatic process is, more or less, closely related to activities of various subtypes of matrix metalloproteinase (MMP) and the corresponding tissue inhibitor of matrix metalloproteinase (TIMP). In general, MMPs facilitate the invasion of the tumour; on the other hand, TIMPs also play an important role in inhibiting MMPs. The TIMP-1 transfected cells or carcinoma cells with an abundant expression of TIMP-1 mRNA inhibit the MMPs' activity to invade the model of basement membranes in various human carcinoma cell lines (Sato et al, 1992; Azuma et al, 1993; Tsuchiya et al, 1993; Belloc et al, 1995; Miyagi et al, 1995).

Recent studies have reported an alternative function of TIMP-1, i.e. as a growth factor; it is highly homologous with erythroid potentiating activity (EPA), which is an autocrine growth factor for the erythroid leukaemia cell line K562 (Docherty et al, 1985; Avalos et al, 1988). Moreover, TIMP-1 also shares homology with a fibroblast elongation factor that is secreted from colon carcinoma cells and which stimulates tumour cell proliferation (Agrez et al, 1995). The TIMP-1 RNA levels were higher in primary colorectal carcinomas with distant metastasis than in those without metastasis (Zeng et al, 1995), and the expression of TIMPs increased with the advance of the neoplastic process (Urbanski et al, 1993). With respect to gastric carcinoma, the enhanced TIMP-1 production in carcinoma tissues was also reported by Nomura et al (1996). Therefore, it is considered important to determine whether or not TIMP-1 may possess a function that accelerates malignant potentiality.

In the present study, we studied the expression of TIMP-1 mRNA in human gastric carcinoma tissue by an RT-PCR assay that enabled us to analyse small sample amounts, such as biopsy specimens, before surgery (Hirokoshi et al, 1992). We examined the correlation between its expression and clinicopathological variables, as well as the significance of its expression as an independent factor to predict the depth of tumour invasion and patient prognosis. We would thus like to emphasize that TIMP-1 expression may be one of the factors indicating the aggressiveness of gastric carcinomas.

### MATERIALS AND METHODS

### Biopsy specimens

Biopsy specimens from 50 patients, including 30 men and 20 women, with gastric carcinoma were available for this study. The average age was 67.5 years with a range from 38 to 87 years. We obtained three and four biopsy specimens from the tumour and normal tissue respectively. Two specimens from tumour tissue and two specimens from normal tissue were used for RNA extraction. The other specimens from the tumour and normal tissue were used for the pathological diagnosis. For tumour biopsy samples, we obtained the tissue from the edge of the primary tumour because the edge area contains both tumour and stromal tissues in the specimen. Consequently, we checked and confirmed that all biopsy specimens stained with haematoxylin and eosin contained tumour epithelial tissue and stromal tissue in the tumour biopsy specimen, and normal epithelial tissue and stromal tissue in the normal biopsy specimen. In most of the above cases, normal biopsy specimens, i.e. specimens obtained from the non-cancerous mucosal tissue, disclosed chronic gastritis with mild to severe stromal fibrosis and inflammatory cell infiltration.

*Received 20 September 1996*
*Revised 28 January 1997*
*Accepted 31 January 1997*

*Correspondence to:* M Mori, Department of Surgery, Medical Institute of Bioregulation, Kyushu University, 4546 Tsurumibaru, Beppu 874, Japan

**Figure 1** (**A**) Autoradiograms show amplicons of tissue inhibitor of matrix metalloproteinase 1 (TIMP-1) produced by reverse transcriptase – polymerase chain reaction (RT-PCR) on serial amounts of RNA from a gastric carcinoma cell line, MKN-7. The linear range of amplification was observed in 1.0 µg of RNA. (**B**) The relationship between the cycle number of PCR and the radioactivity of the amplified product of TIMP-1. The suitable cycle number was 24

### Resected specimens

To examine the discrepancy of the results between biopsy samples and resected samples, we compared both results in ten cases. In this case, the resected specimens were those obtained from the central cut slice of the primary tumour and the normal tissue in the surgically resected stomach.

### Gastric carcinoma cell line for the preliminary experiment

MKN-7, a representative gastric carcinoma cell line, was studied in the preliminary experiments to quantify the amplified PCR product. This cell line was maintained in Dulbecco's modified Eagle medium containing 10% fetal calf serum and antibiotics.

### RNA preparation from tissue specimens

The samples for RNA extraction were immediately stored at –80°C until use. The total RNA was then extracted according to the method of acid guanidinium thiocyanate – phenol – chloroform extraction (Chomczynski and Sacchi, 1987). All samples were treated in Eppendorf tubes (Eppendorf, Germany) and were handled with gloves to avoid contamination with RNAase.

### Reverse transcription

The total RNA of both the tumour and the normal samples from the 50 cases was then reverse transcribed using random hexamer primers into the cDNA as described previously (Mori et al, 1995).

### Oligonucleotide primers of TIMP-1

On the GenBank accession no. S68252, both the sense and antisense primers were synthesized using by an Applied Biosynthesis 394 PCR-Mate DNA Synthesizer. The primer sequences of TIMP-1 were as follows: sense primer (5′-GCCCCTGGCTTCTGGCATCCT-3′) corresponding to human TIMP cDNA 18 to 38 (GenBank) and antisense (5′-GAGGCAGGCAGGCAAGGTGAC-3′) corresponding to human TIMP cDNA 557 to 577 (GenBank). These primers amplified the 559-bp fragment of TIMP-1 cDNA.

### The semiquantitative detection of mRNA

In order to evaluate the amplified product quantitatively by PCR, preliminary experiments were carried out to determine the suitable number of cycles using the linear range of the PCR product in cDNA from MKN-7, a representative gastric carcinoma cell line (Figure 1) (Wang et al, 1989). As a result, the most suitable number of PCR cycles for TIMP-1 was determined to be 24. The acceptable amount of RNA to be reverse transcribed for the amplification ranged from 0.01 to 10 µg because of the linear correlation between the amplified products and the amount of RNA. cDNA corresponding to 1.0 µg of RNA was thus found to be suitable to perform the PCR.

### Polymerase chain reaction

The amplification of TIMP-1 cDNA was performed in a total volume of 25 µl, which included 10× PCR buffer (100 mM Tris-HCl, pH 8.3, 500 mM potassium chloride, 15 mM magnasium

© Cancer Research Campaign 1997

**Figure 2** The samples obtained from three patients were independently assayed two times, starting each time with a new 1-µg sample of RNA. Consequently, the first and the second results were almost similar in each case and showed good reproducibility

**Figure 4** Representative cases of the expression of amplified TIMP-1 in both tumours and normal specimens from patients with gastric carcinoma. The numbers described below indicate the tumour – normal (T/N) ratio of TIMP-1 expression

**Figure 3** Relationship between the T/N ratio of TIMP-1 expression in the resected specimens and in the biopsy specimens from ten representative cases of gastric carcinoma. There was a significant correlation between them ($P$ = 0.0157, $r$ = 0.734)

chloride, 1% Triton X-100), 25 mM dNTP (mixed dATP, dCTP, dGTP and dTTP; each 100 mM), 15 mM TIMP-1 each primer and 1 unit of *Taq* DNA polymerase (Promega, Madison, WI, USA). The antisense primer was end labelled with [γ-$^{32}$P]ATP by using T4 polynucleotide kinase (Takara, Japan). The reactions were subjected to 24 cycles for 1 min at 94°C, 1 min at 60°C and 1 min and 30 s at 72°C. The amplified DNA fragment was electrophoresed on 1.5% agarose gel containing ethidium bromide with a DNA molecular weight marker for comparison. Next, the radioactivities of the amplified products were analysed using a Fuji Image Analyzer (BAS1000, Fuji Photo Film).

### Reproducibility of the experiments

An experiment indicating the reproducibility of the quantification was performed. The samples obtained from three patients were independently assayed two times, starting each time with a new 1-µg sample of RNA.

### Statistical analysis

The association between the clinicopathological variables and the T/N ratio of the TIMP-1 was analysed using Student's $t$-test and Fisher's exact test. In addition, a logistic analysis was also performed to ascertain the independence of the TIMP-1 expression for determining the patient prognosis.

### RESULTS

### Reproducibility of RT-PCR analysis

The first and the second results were almost the same in each case and showed a good reproducibility (Figure 2).

### Comparison of the data from the resected specimens and the biopsy specimens

The T/N ratio of TIMP-1 expression in the resected specimens was 3.84, while that in the biopsy specimens was 3.55. There was a good correlation between the two as shown in Figure 3 ($r$ = 0.734, $P$ = 0.016). These findings suggest that the results of the biopsy specimens reflect those of the resected specimens.

### Analysing the TIMP-1 mRNA levels in tumour and normal tissues

The expression of TIMP-1 mRNA in all 50 cases with gastric carcinoma was observed in both the tumour and the normal samples (Figure 4). The expression of TIMP-1 mRNA could be detected in all biopsy specimens obtained from the normal tissue, and its level varied in each case. We thus considered it to be appropriate to compare the expression level in tumour samples with that in normal samples in each case. The expression level of TIMP-1 was evaluated using the tumour–normal (T/N) ratio of TIMP-1. The average T/N ratio was 7.21, with a range of 85.4 to 0.68. Forty-four cases showed a higher TIMP-1 expression in the tumour specimens than in the normal specimens. Table 1 shows the relationship between the T/N ratio of TIMP-1 mRNA expression and its clinicopathological features using Student's $t$-test.

© Cancer Research Campaign 1997

**Table 1** Clinicopathological variables of the patients studied and the tumour–normal ratio of the tissue inhibitor of metalloproteinase-1 mRNA in gastric carcinomas

| Variables | *n* | TIMP-1 mRNA T/N ratio[a] | *P*-value |
|---|---|---|---|
| Sex | | | NS[b] |
| M | 30 | 6.9 ± 2.4 | |
| F | 20 | 7.7 ± 4.2 | |
| Histology | | | NS |
| Intestinal type | 29 | 7.4 ± 2.9 | |
| Diffuse type | 21 | 7.0 ± 3.5 | |
| Depth of tumour invasion | | | NS (0.077) |
| Within muscularis propria | 29 | 3.9 ± 0.6 | |
| Beyond subserosa | 21 | 11.8 ± 5.0 | |
| Lymph vessel invasion | | | NS |
| Present | 33 | 8.8 ± 3.3 | |
| Absent | 17 | 4.2 ± 0.8 | |
| Vascular vessel invasion | | | NS |
| Present | 12 | 10.2 ± 6.0 | |
| Absent | 38 | 6.3 ± 2.2 | |
| Lymph node metastasis | | | NS |
| Present | 30 | 9.8 ± 3.6 | |
| Absent | 20 | 3.3 ± 0.5 | |
| Stage | | | 0.042 |
| I and II | 30 | 3.6 ± 0.6 | |
| III and IV | 20 | 12.6 ± 5.2 | |
| Prognosis | | | 0.007 |
| Cancer death | 12 | 17.7 ± 8.5 | |
| Alive | 36 | 4.0 ± 0.5 | |
| Other | 2 | 2.3 ± 0.2 | |

[a]Mean ± standard deviation. [b]NS, no significant difference.

Twenty patients belonging to stage III or IV showed a significantly higher T/N ratio of TIMP-1 mRNA than the 30 patients belonging to stage I or II ($P = 0.042$). A significant difference was also observed between the T/N ratio in the 12 patients who died of primary cancer and that of the 36 patients who were still alive at the time of writing ($P = 0.007$). With respect to the depth of tumour invasion, the cases of tumours invading the subserosa or deeper showed a higher T/N ratio than those invading up to the muscularis propria, but it did not reach a statistically significant difference ($P = 0.077$). No significant differences were found between any other clinicopathological features.

The logistic analysis is shown in Table 2. TIMP-1 was the third, and independent, determinant factor for the depth of tumour invasion ($r = 1.748$, $P = 0.038$) (Table 2A). Although TIMP-1 was not an independent prognostic factor ($r = 0.080$, $P = 0.07$), it was the second major prognostic factor after the depth of tumour invasion (Table 2B).

### DISCUSSION

Three TIMP genes (TIMP-1, TIMP-2 and TIMP-3) have so far been identified (Murphy et al, 1981; Stettler-Stevenson et al, 1989; Palvoff et al, 1992). TIMP-1 possesses 41% amino acid homology with TIMP-2. TIMP-1 is a Mr 28 000 glycoprotein and binds to MMPs in a 1:1 molar ratio and specifically inhibits MMP activities (Welgus et al, 1981). TIMP-1 forms a complex with proMMP9 and inhibits the activation of proMMP9 by stromelysin (Goldberg et al, 1992); inhibition of activity is the result of binding the active site of the MMP (Birkedal-Hansen et al, 1993; Denhardt et al, 1993).

During the invasion and metastatic progression of the carcinoma cells, TIMPs have been reported to be a negative regulator of MMPs in human and mouse tumour models in vitro and in vivo. The TIMP-1 antisense transfected Swiss 3T3 cell lines reduced the invasive activity with a lower expression of TIMP-1 (Khokha et al, 1989). TIMP-1 gene transfected gastric carcinoma cells have been reported to reduce the ability of invasion or metastasis (Tsuchiya et al, 1993). Several previous studies have revealed that highly malignant tumours produce both higher MMPs and lower TIMPs, while less malignant tumours successfully inhibit the activities of MMPs by TIMP activity (Sato et al, 1992; Azuma et al, 1993; Miyagi et al, 1995; Mohanam et al, 1995).

However, in another study using clinical samples, the expression of TIMP mRNA was higher in carcinoma tissue. In studies of various carcinoma cases, such as stomach, colorectal, head and neck, and pancreas, both MMPs and TIMPs were found to correlate with an increased metastatic and invasive potential of tumour cells (Polette et al, 1993; Urbanski et al, 1993; Gress et al, 1995; Zeng et al, 1995; Nomura et al, 1996). Zeng et al (1995) reported that higher TIMP-1 RNA levels were found in colorectal carcinoma with metastasis than in those without metastasis. Urbanski et al (1993) reported that the expression of TIMP mRNA thus paralleled the expression of MMPs and was also highly intercorrelated with the neoplastic process of human sporadic colorectal carcinomas. We demonstrated in this gastric carcinoma study that the expression of TIMP-1 RNA was an independent factor influencing

**Table 2**

| Variables | Regression coefficient | Standard error | Odds ratio (95% confidence interval) | *P*-value |
|---|---|---|---|---|
| (A) *Multivariate analysis on the determination of the depth of tumour invasion (logistic analysis)* | | | | |
| Lymphatic vessel invasion | 2.897 | 1.20 | 18.1 (1.63–201) | 0.002 |
| Vascular vessel invasion | 1.803 | 0.93 | 6.07 (0.934–39.4) | 0.035 |
| TIMP-1 | 1.748 | 0.911 | 5.74 (0.918–35.9) | 0.038 |
| (B) *Multivariate analysis on the determination of the patient prognosis[a] (logistic analysis)* | | | | |
| Depth of tumour invasion | 2.45 | 0.884 | 11.6 (1.95–68.8) | 0.002 |
| TIMP-1 | 0.08 | 0.09 | 1.08 (0.903–1.30) | 0.07 |

[a]Two patients who died during the operation were excluded from the analysis.

© Cancer Research Campaign 1997

the depth of tumour invasion and the second determinant factor for patient prognosis after the depth of tumour invasion.

A discrepancy still exists however between the function of TIMP-1 as an inhibitor of tumour cell invasion in vitro and the higher expression of TIMP-1 in human carcinoma cells, according to previous reports or our findings. There are several possible explanations for this discrepancy. First of all, a higher expression of MMPs was observed in tissues with invasive carcinoma cells, which induce macrophages with cytokines and thus elevate the expression of TIMPs (Campbell et al, 1991; Lotz and Guerne, 1991; Partridge et al, 1993; Richards and Agro, 1994). Second, as suggested by several investigators, TIMP-1 has two distinct activities, i.e. a metalloproteinase inhibitory activity and a growth factor activity (Avalos et al, 1988; Agrez et al, 1995; Chesler et al, 1995).

Recent advances in the techniques of molecular biology have enabled clinicians to obtain both more detailed and more objective information on tumour biology. Such advances include the clarification of multiple-step carcinogenesis in gastrointestinal carcinomas; molecular-level diagnoses, such as the detection of micrometastasis in lymph nodes (Mori et al, 1995), bone marrow or peripheral blood samples (Mori et al, 1996); or molecular-level new therapies, such as gene therapy. A new, molecular-level prognostic factor still needs to be established that can be widely used in the practical follow-up of patients (Mori et al, 1993*a* and *b*). RT-PCR assays enable the molecular biological analysis of small samples. Some problems exist, however, when performing such analyses on small biopsy samples. The first is the problem of reproducibility. In this study, we confirmed that RT-PCR for the TIMP-1 gene showed almost the same results when the experiments were repeated. The second problem is whether or not the biopsy specimens accurately reflect the whole material of the resected specimens. We compared the RT-PCR results obtained from biopsy specimens with those from resected specimens. The results consequently demonstrated a significantly good correlation. We thus consider that, at least for the TIMP-1 gene, the results obtained from the biopsy specimens accurately reflect those obtained from resected specimens of the primary tumour, and we believe that useful preoperative information on molecular-level factors can be achieved by the molecular examination of biopsy specimens.

The present study demonstrated that the evaluation of TIMP-1 expression using an RT-PCR assay with biopsy specimens may therefore be useful in predicting the aggressive behaviour of gastric carcinomas. Such an assay is objective and can be done preoperatively, and therefore it is considered to be useful in a clinical setting.

### ACKNOWLEDGEMENTS

We would like to thank Dr Kouhei Akazawa and Ms Junko Tsuchihashi for their assistance in the data analysis.

### REFERENCES

Agrez MV, Meldrum CJ, Sim AT, Aebersold RH, Clark IM, Cawston TE and Burns GF (1995) A fibroblast elongation factor purified from colon carcinoma cells shares sequence identity with TIMP-1. *Biochem Biophys Res Commun* **206**: 590–600

Avalos B, Kaufman S, Tomonaga M, Williams R, Golde D and Gasson J (1988) K562 cells produce and respond to human erythroid-potentiating activity. *Blood* **71**: 1720–1725

Azuma M, Tamatani T, Fukui K, Yoshida H, Kamogashira T, Ogino K, Suzuki T and Sato M (1993) Role of plasminogen activators, metalloproteinases and the tissue inhibitor of metalloproteinase-1 in the metastatic process of human salivary-gland adenocarcinoma cells. *Int J Cancer* **54**: 669–676

Belloc C, Lu, H, Soria C, Fridman R, Legrand Y and Menashi S (1995) The effect of platelets on invasiveness and protease production of human mammary tumor cells. *Int J Cancer* **60**: 413–417

Birkedal-Hansen H, Moore W, Bodden M, Windsor L, Birkedal-Hansen B, DeCarlo A and Engler J (1993) Matrix metalloproteinases: a review. *Crit Rev Oral Biol Med* **4**: 197–250

Campbell EJ, Cury JD, Shapiro SD, Goldberg GI and Welgus HG (1991) Neutral proteinases of human mononuclear phagocytes. Cellular differentiation markedly alters cell phenotype for serine proteinases, metalloproteinases, and tissue inhibitor of metalloproteinases. *J Immunol* **146**: 1286–1293

Chesler L, Golde DW, Bersch n. and Johnson MD (1995) Metalloproteinase inhibition and erythroid potentiation are independent activities of tissue inhibitor of metalloproteinase-1. *Blood* **86**: 4506–4515

Chomczynski P and Sacchi N (1987) Single-step method of RNA isolation by acid guanidinium thiocyanate-phenol chloroform extraction. *Anal Biochem* **162**: 156–159

Denhardt D, Feng B, Edwards D, Cocuzzi E and Malyankar U (1993) Tissue inhibitor of metalloproteinases (TIMP, aka EPA): structure, control of expression and biological functions. *Phamacol Ther* **59**: 329–341

Docherty A, Lyons A, Smith B, Wright E, Stephens P, Harris T, Murphy G and Reynolds J (1985) Sequence of human tissue inhibitor of metalloproteinase and its identity to erythroid-potentiating activity. *Nature* **318**: 66–69

Goldberg GI, Strongin A, Collier IE, Genrich LT and Marmer BL (1992) Interaction of 92-kDa type IV collagenase with the tissue inhibitor of metalloproteinases prevents dimerization, complex formation with interstitial collagenase, and activation of the proenzyme with stromelysin. *J Biol Chem* **267**: 4583–4591

Gress TM, Muller PF, Lerch MM, Friess H, Buchler M and Adler G (1995) Expression and in-situ localization of genes coding for extracellular matrix proteins and extracellular matrix degrading proteases in pancreatic cancer. *Int J Cancer* **62**: 407–413

Hirokoshi T, Danenberg KD, Stadbauer THW, Volkenandt M, Shea, LCC, Aigner K, Gustavsson B, Leichman L, Frosing R, Ray M, Gibson NW, Spears CP and Dannanberg PV (1992) Quantitation of thymidylate synthease, dihydrofolate reductase, and DT-diaphorase gene expression in human tumors using the polymerase chain reaction. *Cancer Res* **52**: 108–116

Khokha R, Waterhouse P, Yagel S, Lala P, Overall C, Norton G and Denhardt D (1989) Antisense RNA-induced reduction in murine TIMP levels confers oncogenicity of Swiss 3T3 cells. *Science* **243**: 947–950

Lotz M and Guerne PA (1991) Interleukin-6 induces the synthesis of tissue inhibitor of metalloproteinases-1/erythroid potentiating activity (TIMP-1/EPA). *J Biol Chem* **266**: 2017–2020

Miyagi E, Yasumitsu H, Hirahara F, Minaguchi H, Koshikawa N, Miyazaki K and Umeda M (1995) Characterization of matrix-degrading proteinases and their inhibitors secreted by human gynecological carcinoma cells. *Jpn J Cancer Res* **86**: 568–576

Mohanam S, Wang SW, Rayford A, Yamamoto M, Sawaya R, Nakajima M, Liotta LA, Nicolson GL, Stettler-Stevenson W and Rao JS (1995) Expression of tissue inhibitors of metalloproteinases: negative regulators of human glioblastoma invasion in vivo. *Clin Exp Met* **13**: 57–62

Mori M, Barnard GF, Staniunus RJ, Jessup JM, Steele GD and Chen LB (1993*a*) Prothymosin-α mRNA expression correlates with that of c-myc in human colon cancer. *Oncogene* **8**: 2821–2826

Mori M, Staniunus RJ, Barnard GF, Jessup JM, Steele GD and Chen LB (1993*b*) The significance of carbonic anhydrase expression in human colorectal cancer. *Gastroenterology* **105**: 820–826

Mori M, Mimori K, Inoue H, Barnard GF, Tsuji K, Nanbara S, Ueo H and Akiyoshi T (1995) Detection of cancer micrometastasis in lymph nodes by reverse transcription-polymerase chain reaction. *Cancer Res* **55**: 3417–3420

Mori M, Mimori K, Ueo H, Karimine N, Barnard GF and Sugimachi K (1996) Molecular detection of circulating solid carcinoma cells in the peripheral blood: the concept of early systemic disease. *Int J Cancer* **68**: 739–743

Murphy G, Cawston T and Reynolds J (1981) An inhibitor of collagenase from human amniotic fluid. Purification, characterization and action on metalloproteinases. *Biochemistry* **195**: 167–170

Nomura H, Fujimoto N, Seiki M, Mai M and Okada Y (1996) Enhanced production of matrix metalloproteinases and activation of matrix metalloproteinase 2 (gelatinase A) in human gastric carcinomas. *Int J Cancer* **69**: 9–16

Palvoff N, Staskus P, Kishnani N and Hawkes S (1992) A new inhibitor of metalloproteinases from chicken: ChIMP-3. A third member of the TIMP family. *J Biol Chem* **267**: 17321–17326

© Cancer Research Campaign 1997

Partridge CA, Jeffrey JJ and Malik AB (1993) A 96-kDa gelatinase induced by TNF-alpha contributes to increased microvascular endothelial permeability. *Am J Physiol* **265**: 438–447

Polette M, Clavel C, Birembaut P and De CY (1993) Localization by in situ hybridization of mRNAs encoding stromelysin 3 and tissue inhibitors of metallo-proteinases TIMP-1 and TIMP-2 in human head and neck carcinomas. *Pathol Res Pract* **189**: 1052–1057

Richards CD and Agro A (1994) Interaction between oncostatin M, interleukin 1 and prostaglandin E2 in induction of IL-6 expression in human fibroblasts. *Cytokine* **6**: 40–47

Sato H, Kida Y, Mai M, Endo Y, Sasaki T, Tanaka J and Seiki M (1992) Expression of genes encoding type IV collagen-degrading metalloproteinases and tissue inhibitors of metalloproteinases in various human tumor cells. *Oncogene* **7**: 77–83

Stettler-Stevenson W, Krutzsch H and Liotta L (1989) Tissue inhibitor of metalloproteinase (TIMP-2). *J Biol Chem* **264**: 17374–17378

Tsuchiya Y, Sato H, Endo Y, Okada Y, Mai M, Sasaki T and Seiki M (1993) Tissue inhibitor of metalloproteinase 1 is a negative regulator of the metastatic ability of a human gastric cancer cell line, KKLS, in the chick embryo. *Cancer Res* **53**: 1397–1402

Urbanski SJ, Edwards DR, Hershfield N, Huchcroft SA, Shaffer E, Sutherland L and Kossakowska AE (1993) Expression pattern of metalloproteinases and their inhibitors changes with the progression of human sporadic colorectal neoplasia. *Diag Mol Pathol* **2**: 81–89

Wang A, Doyle V and Mark D (1989) Quantitation of mRNA by the polymerase chain reaction. *Proc Natl Acad Sci USA* **86**: 9717–9721

Welgus HG, Jeffrey JJ and Eisen AZ (1981) The collagen substrate specificity of human skin fibroblast collagenase. *J Biol Chem* **256**: 9511–9515

Zeng Z-S, Cohen AM, Zahng Z-F, Stetler-Stevenson W and Guillem JG (1995) Elevated tissue inhibitor of metalloproteinase 1 RNA in colorectal cancer stroma correlates with lymph node and distant metastasis. *Clin Cancer Res* **1**: 899–906

© Cancer Research Campaign 1997